# *Acidovorax citrulli* type III effector AopU interferes with plant immune responses and interacts with a watermelon E3 ubiquitin ligase

**DOI:** 10.3389/fmicb.2023.1275032

**Published:** 2023-10-09

**Authors:** Linlin Yang, Mei Zhao, Xiaoxiao Zhang, Jie Jiang, Nuoya Fei, Weiqin Ji, Yunfeng Ye, Wei Guan, Yuwen Yang, Tingchang Zhao

**Affiliations:** ^1^Department of Plant Pathology, Plant Protection College, Shenyang Agricultural University, Shenyang, China; ^2^State Key Laboratory for Biology of Plant Diseases and Insect Pests, Institute of Plant Protection, Chinese Academy of Agricultural Sciences, Beijing, China; ^3^Department of Plant Pathology, College of Plant Protection, China Agricultural University, Beijing, China; ^4^Horticultural Research Institute, Guangxi Academy of Agricultural Sciences, Nanning, Guangxi, China; ^5^National Nanfan Research Institute (Sanya), Chinese Academy of Agricultural Sciences, Sanya, China

**Keywords:** *Acidovorax citrulli*, type III effector, AopU, E3 ubiquitin ligase, plant immunity

## Abstract

*Acidovorax citrulli* is a seed-borne bacterium that causes bacterial fruit blotch of watermelon and other cucurbit plants worldwide. It uses a type III secretion system to inject type III effectors (T3Es) into plant cells, which affect the host immune responses and facilitate pathogen colonization. However, the current understanding of the specific molecular mechanisms and targets of these effectors in *A. citrulli* is limited. In this study, we characterized a novel T3E called AopU in *A. citrulli* group II strain Aac5, which shares homology with XopU in *Xanthomonas oryzae*. The *Agrobacterium*-mediated gene transient expression system was used to study the effect of AopU on host immunity. The results showed that AopU localized on the cell membrane and nucleus of *Nicotiana benthamiana*, inhibited reactive oxygen species burst induced by flg22 and the expression of marker genes associated with pathogen-associated molecular pattern-triggered immunity, but activated salicylic acid and jasmonic acid signal pathways. Further investigations revealed that AopU interacts with E3 ubiquitin ligase ClE3R in watermelon, both *in vitro* and *in vivo*. Interestingly, the deletion of *aopU* did not affect the virulence of *A. citrulli*, suggesting that AopU may have functional redundancy with other effectors in terms of its role in virulence. Collectively, these findings provide new insights into the mechanism of plant immune responses regulated by *A. citrulli* T3Es.

## 1. Introduction

In nature, a never-ending arms race exists between host plants and pathogenic microorganisms. Pathogens employ various tactics to infect and propagate, while plants, in turn, activate their defense mechanisms to counter these attacks. This perpetual competition between pathogens and crops leads to a continuous escalation of both offensive and defensive strategies. Over time, pathogens have developed a diverse arsenal of weapons, while plants have evolved increasingly sophisticated defense systems to protect themselves. Consequently, a dynamic equilibrium is gradually formed between the virulence of pathogens and the resistance of plants (Ngou et al., 2022; Zhang et al., 2022).

Plants have two innate immune systems to protect themselves against pathogens. The first layer of defense is pathogen-associated molecular pattern (PAMP)-triggered immunity (PTI), mediated by the pattern recognition receptors (PRRs; Jones and Dangl, 2006). The PRRs are located on plant cell membranes and specifically recognize conserved PAMPs, such as flagellin, elongation factor Tu (EF-Tu), peptidoglycan (PGN), and chitin (Gómez-Gómez et al., 1999; Zipfel et al., 2006; Boller and Felix, 2009). Once these PAMPs are recognized, they induce a series of defensive responses, including the production of reactive oxygen species (ROS), callose deposition, and activation of defense-related genes (Gómez-Gómez et al., 1999). Currently, some well-known PRRs include FLS2, which recognizes the N-terminal 22-amino acid peptide (flg22) of flagellin in *Arabidopsis*; EFR, which recognizes the N-terminal 18-amino acid peptide (elfl8) of EF-Tu in *Arabidopsis* (Gómez-Gómez and Boller, 2000; Zipfel et al., 2006); CERK1, the chitin elicitor-binding kinase in *Arabidopsis*, and the chitin elicitor-binding protein in rice, CEBiP (Kaku et al., 2006; Miya et al., 2007). In addition, Liu et al. (2012) found that LYP4 and LYP6 in rice function as PRRs of both bacteria and fungi, recognizing bacterial PGN and fungal chitin.

The second layer of defense in plants, known as effector-triggered immunity (ETI), plays a crucial role in combating pathogenic infections. The largest family of intracellular resistance proteins (R proteins), nucleotide-binding domain and leucine-rich repeat receptors (NLRs) recognize pathogen effectors (Avirulence proteins, Avr). This recognition then activates plant immune responses (Jones and Dangl, 2006; Dodds and Rathjen, 2010). ETI is more pronounced compared to PTI. It is often accompanied by a strong hypersensitive response (HR), which can lead to programmed cell death (PCD) around the infected site. As a result, this limits further infestation by pathogenic microorganisms (Ausubel, 2005; Chisholm et al., 2006; Jacob et al., 2013). Recognition of effectors by NLRs can occur through direct or indirect interactions (Cui et al., 2015). For example, Pi-ta directly interacts with *Magnaporthe oryzae* effector AVR-Pita (Jia et al., 2000), while SUMM2 detects the *Pseudomonas syringae* effector HopAI1 by monitoring the activity of the MEKK1-MKK1/MKK2-MPK4 cascade pathway (Zhang et al., 2012). Recent research has revealed that PTI and ETI are not independent of each other but rather synergistic, as supported by increasing evidence (Ngou et al., 2021; Yuan et al., 2021).

To successfully invade host plants, pathogens have developed various strategies to subvert the plants defense responses. One common strategy employed by plant pathogenic bacteria is to secrete effector proteins into plant cells through the type III secretion system (T3SS; Büttner and He, 2009). Several type III effectors (T3Es) produced by *P. syringae*, such as AvrPto, AvrPtoB, AvrPphB, HopAO1, HopF2, and HopB1 interfere with PTI by inhibiting the activation of PRR complexes (Zhou et al., 2014; Li et al., 2016). In addition to PTI, *P. syringae* T3Es HopZ3, AvrPphB, AvrRpt2, HopBF1, and *Xanthomonas* T3E AvrBsT can suppress ETI by blocking the recognition of Avr proteins by R proteins (Schreiber et al., 2021). Besides, T3Es can affect the plant’s immune responses by interfering with phytohormone signaling pathways or host modification systems. For example, *P. syringae* T3E HopZ1a promotes the degradation of JAZ protein and activates the jasmonic acid (JA) pathway to facilitate infection (Jiang et al., 2013). Another example is the T3E XopP_Xoo_ from *X. oryzae* pv. *oryzae* (*Xoo*), which negatively regulates the immune responses of rice by inhibiting the E3 ubiquitin ligase activity of OsPUB44 (Ishikawa et al., 2014). By secreting effector proteins, pathogens are able to manipulate the plant’s defense mechanisms and establish successful infections. Understanding the mechanisms used by pathogens to subvert plant immunity is crucial for developing effective strategies to protect crops from devastating diseases.

Seed-borne bacterial fruit blotch (BFB) is a devastating plant disease that primarily affects watermelon, melon, and other cucurbit crops. It has caused huge economic losses to the cucurbit industry worldwide (Burdman and Walcott, 2012). The disease is caused by *Acidovorax citrulli*, which can be divided into at least two genetically distinct groups. Group I strains are mainly isolated from non-watermelon hosts, and showed little variation in virulence to cucurbit hosts. However, group II strains are predominantly isolated from watermelon and highly virulent to watermelon (Walcott et al., 2000; Yang et al., 2019). Currently, there are no commercially available resistant varieties for *A. citrulli*, and effective means for the prevention and control of BFB are still lacking.

Understanding the virulence mechanism of *A. citrulli* in watermelon is crucial for the development of effective control strategies. The T3SS is essential for *A. citrulli* to cause disease in host plants and induce an HR in nonhost plants (Bahar and Burdman, 2010). Moreover, the hypersensitive response and pathogenicity (*hrp*) gene cluster encoding the T3SS in *A. citrulli* is similar to those found in *Xanthomonas* species and *Ralstonia solanacearum*, belonging to class II (Büttner and Bonas, 2002). In *A. citrulli* group II strain Aac5, the T3SS regulatory genes *hrpG* and *hrpX*, as well as the T3SS apparatus gene *hrpE* are critical for pathogenicity (Wang et al., 2016; Zhang et al., 2018; Ji et al., 2022). Furthermore, *hrpG* and *hrpX* play important roles in regulating T3Es. Three T3Es AopN, AopP, and AopV in Aac5 have been successively identified, and their regulation is mediated by *hrpG* and *hrpX*. These effectors all interfered with plant immune response. AopN inhibited ROS burst but induced programmed cell death (PCD) in *Nicotiana benthamiana*. AopP modulated salicylic acid (SA) signaling to suppress plant immunity by interacting with the watermelon transcription factor WRKY6. AopV inhibited plant immunity by targeting aromatic dehydratase ADT6 (Zhang et al., 2020a,b; Jiang et al., 2022). Furthermore, some effector proteins were also screened using *hrpX* mutant in the group I strain M6 (Jiménez-Guerrero et al., 2020). In fact, *A. citrulli* group I and II strains have distinct different repertoires of T3Es, which may contribute to their host preference (Yan et al., 2017).

In recent years, some progress has been made in understanding the T3Es in *A. citrulli*, but there is still a lack of knowledge about how T3Es participate in the interaction between *A. citrulli* and its host. In this study, we identified a new T3E AopU in *A. citrulli* strain Aac5. To investigate the impact of AopU on plant immune responses, we used *N*. *benthamiana*, a commonly used model plant for studying effector protein function and a known host of *A. citrulli* (Pitino et al., 2018; Wei et al., 2018; Fang et al., 2019; Traore et al., 2019). Our findings revealed that AopU can inhibit the ROS burst and the expression of PTI marker genes, but activate the SA and JA pathways. Moreover, the interaction between AopU and the putative E3 ubiquitin ligase ClE3R in the host watermelon was verified through yeast two-hybrid, luciferase complementation assay (LCA), and glutathione S-transferase (GST) pull-down assays. This study provides new insights into the mechanism through which *A. citrulli* T3Es regulate plant immune responses.

## 2. Materials and methods

### 2.1. Plant materials and culture conditions

Watermelon plants (cv. “Ruixin”) for virulence assay were grown in a climatic chamber with relative humidity (RH) of 40%–60%, and 28°C, 16 h light/25°C, and 8 h dark for 2 weeks. The *N. benthamiana* plants for transient expression assays and *N. tabacum* var. *Samsun* plants for HR assays were grown in a climatic chamber with RH of 60% and 25°C, 16 h light/ 22°C, and 8 h dark for 3–4 weeks.

### 2.2. Bacterial strains and growth conditions

All *A. citrulli* strains were cultured at 28°C in King’s B (KB) medium for inoculation assays or T3SS induction broth XVM2 to induce effector expression (Wengelnik et al., 1996). *Escherichia coli* DH5α strains were grown in Lysogeny Broth (LB) medium at 37°C and *Agrobacterium tumefaciens* GV3101 strains were grown in LB medium at 28°C. For the solid medium, agar (10 g/L) was added to the above liquid medium. When required, media were supplemented with ampicillin (Amp, 100 μg/mL), kanamycin (Kan, 50 μg/mL), chloramphenicol (Cm, 25 μg/mL), and rifampicin (Rif, 50 μg/mL). All strains and plasmids used in this study are listed in Supplementary Table S1. DH5α competent cells were purchased from Tiangen (Beijing, China), and GV3101 competent cells were purchased from Biomed (Beijing, China).

### 2.3. Sequence analysis of AopU

The DNA or amino acid sequences used in this study were searched in the National Center for Biotechnology Information (NCBI) database.^1^ Primers were designed based on the gene *Aave_0114* in *A. citrulli* genome AAC00-1 (GenBank accession number CP000512.1), which encodes *aopU*. All primers used in this study are listed in Supplementary Table S2. DNAMAN version 5.2.2 was used for amino acid sequence alignment. Additionally, the predicted structure and function of AopU were analyzed using UniProt.^2^

### 2.4. Assay for the determination of promoter activity

The promoter sequence of *aopU* was cloned from strain Aac5 using KOD-Plus-Neo (TOYOBO, Osaka, Japan) by PCR and constructed into the vector pBBRNolacGUS modified by Zhang et al. (2018) to generate pBBR-*aopU*-GUS. Then pBBR-*aopU*-GUS was transferred into wild-type strain Aac5, *hrpG* mutant strain Δ*hrpG*, and *hrpX* mutant strain Δ*hrpX* to construct WT-*aopU*-GUS, Δ*hrpG*-*aopU*-GUS, and Δ*hrpX*-*aopU*-GUS through tri-parental mating. The promoter activity was detected as previously described (Zhang et al., 2013). This assay was conducted three times.

### 2.5. Western blotting analysis

The secretory function of AopU was determined using western blotting as previously described with slight modifications (Sun et al., 2011; Zhang et al., 2018). The *aopU* open reading frame (ORF) and its promoter sequence were amplified using KOD-Plus-Neo and inserted into the vector pBBRNolac-4FLAG by ClonExpress II One Step Cloning Kit (Vazyme Biotech, Nanjing, China). The recombinant vector was then introduced into the wild-type strain Aac5 and *hrcJ* mutant strain Δ*hrcJ* through tri-parental mating. The strains were incubated until they reached an optical density (OD) of 0.8 at 600 nm in XVM2 medium. Then, 1 mL of bacterial solution was centrifuged at 12,000 g for 5 min at 4°C. The supernatant was discarded, and 200 μL 4× Laemmli protein sample buffer (Bio-Rad, California, United States) was added to resuspend the bacteria as intracellular samples. The samples were then denatured by heating for 10 min at 95°C. The supernatant was collected by centrifugating the remaining bacterial solution at 4,000 g for 10 min at 4°C, and then filtered through a 0.45 μm filter. The supernatant was precipitated with 10% trichloroacetic acid for 2–3 h, then centrifuged at 4°C for 20 min at 12,000 g. After the supernatant was discarded, the precipitate was suspended with 1 mL of precooled anhydrous ethanol, and the cells were collected by centrifugation at 20,000 g at 4°C. The previous step was repeated, and 200 μL 4× Laemmli protein sample buffer was added to resuspend the precipitate as extracellular protein. To denature the protein, it was treated at 95°C for 10 min. Intracellular and extracellular samples were analyzed using immunoblotting with the FLAG antibody (anti-DDDDK-tag mAb-HRP-DirecT; 1:5,000 dilution; MBL, Beijing, China). Glyceraldehyde 3-phosphate dehydrogenase (GAPDH) antibody was used as the internal reference protein (1:2,500 dilution; Abclonal, Wuhan, China). The experiment was conducted three times.

### 2.6. Construction of *aopU* marker-less deletion mutant and its complement strain in *Acidovorax citrulli* Aac5

To construct the *aopU* mutant Δ*aopU*, a 601 bp fragment upstream of the *aopU* ORF and a 534 bp fragment downstream were amplified using KOD-Plus-Neo. The two fragments were fused using gene splicing by overlap extension (SOEing), and then ligated into the pk18mobsacB vector to create the plasmid pK18-*aopU*LR. Using the helper plasmid pRK600, the recombinant vector pK18-*aopU*LR was introduced into *A. citrulli* strain Aac5 by tri-parental mating. The *aopU* deletion mutant was generated by homologous recombination. The screening and verification processes were conducted following the protocols described in previous studies (Zhang et al., 2018; Ji et al., 2022).

To construct complement strain Δ*aopU*-comp, the full-length *aopU* gene and its native promoter were inserted into the vector pBBRNolac-4FLAG (Zhang et al., 2018). The recombinant vector pBBR-*aopU*-4FLAG was then introduced into the Δ*aopU* strain through tri-parental mating. Furthermore, to eliminate the effect of the vector, the empty vector pBBRNolac-4FLAG was transferred into the Aac5 and Δ*aopU* to generate strain Aac5-pBBR and Δ*aopU*-pBBR (Fei et al., 2022). The screening and verification processes were conducted as described in previously (Qiao et al., 2022; Liu et al., 2023).

### 2.7. Virulence and *in planta* bacterial population assays

Watermelon seedlings were inoculated to determine the role of *aopU* on the virulence of Aac5. The protocol was as described by Ren et al. (2014) with few modifications. Strains Aac5-pBBR, Δ*aopU*-pBBR, and Δ*aopU*-comp were cultured in KB liquid medium, centrifuged at 5,000 rpm for 10 min at room temperature, and resuspended in sterilized distilled water to a concentration of 1 × 10^4^ CFU/mL. The cotyledons of 2-week-old watermelon seedlings were inoculated with the bacterial suspension using 1 mL syringes. The inoculated seedlings were then incubated in a climatic chamber. As negative controls, sterile water injection was used. The samples were taken and photographed at 4, 24, 48, 72, 96, and 120 h post inoculation (hpi). The bacterial populations were then measured on inoculated watermelon cotyledons collected at 4, 24, and 48 hpi. The watermelon cotyledons were surface sterilized in 75% ethanol for 1 min and rinsed three times with sterile distilled water. Then the leaf discs (9 mm in diameter) were taken and homogenized in sterile water. Each treatment contains six leaf discs, and there were three treatments. The resulting homogenate was serially diluted in a 10-fold gradient. Then 10 μL of each dilution were plated on KB agar plates with antibiotics (Amp and Kan). The plates were then incubated at 28°C for 48 h. After the incubation period, colonies were counted to determine the bacterial populations. The experiment was conducted three times.

### 2.8. HR assays in *Nicotiana tabacum*

Strains Aac5-pBBR, Δ*aopU*-pBBR, and Δ*aopU*-comp cultured to the logarithmic growth phase were centrifuged at room temperature and then resuspended with sterilized water. Three-week-old *N. tabacum* leaves were inoculated with bacterial suspension at OD_600_ = 0.3. Plants infiltrated with sterilized water were used as negative controls. All treatments were infiltrated into different positions of the same leaf. For each experiment, three tobacco plants were treated. HR was examined at 24 hpi. The experiment was conducted three times.

### 2.9. *Agrobacterium*-mediated transient expression assays in *Nicotiana benthamiana*

The *aopU* ORF was inserted into the pYBA1132-eGFP vector (C-terminal of AopU fused with an eGFP tag), and then transformed into *A. tumefaciens* GV3101. The empty vector (EV) pYBA1132-eGFP transferred into GV3101 served as a control. The *A. tumefaciens* strains were grown in LB liquid containing kanamycin and rifampicin for 24 h and then centrifuged at 28°C and 4,000 rpm for 10 min. The bacterial pellet was resuspended and adjusted to OD_600_ = 0.5 using a buffer solution (10 mM 2-Morpholinoethanesulphonic acid (MES, pH = 5.6), 100 μM acetosyringone (As), and 10 mM MgCl_2_). The bacterial suspension was incubated at room temperature in the dark for 3–4 h. The *N. benthamiana* plants were inoculated with the bacterial suspension using 1 mL syringes and incubated in a chamber for 48 h (Traore and Zhao, 2011).

### 2.10. Assay for ROS detection

For quantitative ROS detection, the EV and AopU were transiently expressed in *N. benthamiana* and sampled at 48 hpi. Three independent plants were used for each treatment, and 12 leaf discs were punched and placed in separate wells of a 96-well white polystyrene microplate (Corning, New York, USA), with each well initially filled with 100 μL sterile water before sample addition. After 16 h of incubation, the sterile water was discarded, and 100 μL of elicitor mixture containing 100 nM flg22 (Genscript, New Jersey, United States), 20 μg/mL horseradish peroxidase (Sigma, Saint Louis, United States), and 100 μM luminol (Sigma, Saint Louis, United States) was added to each well. The plate was then immediately placed into a multimode plate reader (Tecan Infinite F200, Switzerland) to record ROS production and monitored at 2 min intervals for 60 min. The protocol was as described by Sang and Macho (2017) with few modifications. This assay was performed three times.

For qualitative ROS detection, strains Aac5-pBBR, Δ*aopU*-pBBR, and Δ*aopU*-comp were injected into watermelon cotyledons at OD_600_ = 0.3, and sampled at 24 hpi. The hydrogen peroxide was detected using 3, 3′-diaminobenzidine (DAB) staining as previously described (Daudi and O'Brien, 2012). Three independent plants were used for each treatment and this assay was performed three times.

### 2.11. SA and JA content assays

For SA or JA assessment, the EV and AopU were transiently expressed in *N. benthamiana*, and samples were taken at 48 hpi. The SA or JA levels in leaves were measured by high-performance liquid chromatography (HPLC) as previously described (Zhang et al., 2020a). Three independent plants were used for each treatment and the experiment was conducted three times with three technical replicates each time.

### 2.12. RNA extraction and real-time quantitative PCR

To determine whether *aopU* was regulated by *hrpG* and *hrpX*, Aac5, Δ*hrpG*, and Δ*hrpX* were cultured in XVM2 medium to OD_600_ = 0.6 and the total RNA of each strain was extracted using the Trizol method. cDNA was synthesized using the FastKing One Step Genome Removal and cDNA Strand Synthesis Premixed Reagent Kit (Tiangen, Beijing, China) with 2 mg RNA used as a template. The synthesized cDNA from Aac5, Δ*hrpG*, and Δ*hrpX* were used as templates for RT-qPCR using the SuperReal PreMix Plus (SYBR Green) Kit (Tiangen, Beijing, China). The real-time quantitative PCR (RT-qPCR) analysis was performed on an Applied Biosystems 7500 instrument (ABI, Waltham, Massachusetts, United States). The relative expression of *aopU* was calculated using the 2^−∆∆CT^ method (Livak and Schmittgen, 2001), and *rpoB* was used as the reference gene.

To detect the effect of AopU on the expression of PTI marker genes, the EV and AopU were transiently expressed, and sampled at 48 hpi. Six disks from three independent plants were taken for each treatment. Subsequently, *N. benthamiana* leaves were induced with 100 nM flg22 for 30 min, following which total RNA was extracted. The relative expression of *NbPti5*, *NbAcre31*, and *NbGras2* was determined as described above with *EF1α* as the reference gene (Nguyen et al., 2010).

To detect the effect of AopU on the expression of SA marker genes (*NbICS1*, *NbPR1*, and *NbPAL05*) and JA marker genes (*NbLOX2* and *NbAOS*; Sang et al., 2020), the total RNA of *N. benthamiana* leaves expressing EV or AopU was extracted. The RT-qPCR procedure mentioned earlier was followed for gene expression analysis. All the above experiments were conducted three times with three technical replicates.

### 2.13. Subcellular localization of AopU in *Nicotiana benthamiana*

AopU was transiently expressed in *N. benthamiana*, and EV served as a control. Three independent plants were used for each treatment. The subcellular localization of AopU was observed using laser confocal microscopy (Carl Zeiss LSM 880, Jena, Germany). The green fluorescent signal produced by AopU-eGFP was observed at 488 nm excitation wavelength, and the red fluorescence signal produced by nuclear localization marker protein H2B-RFP was observed at 588 nm excitation wavelength. This assay was performed three times.

### 2.14. Yeast two-hybrid assay

The *aopU* coding sequence was inserted into the pGBKT7 vector to generate pGBKT7-AopU, and the full-length cDNA of *ClE3R* was inserted into the pGADT7 vector to generate pGADT7-ClE3R. Then, pGBKT7-aopU and pGADT7-ClE3R were co-transformed into the Y2H Gold strain. The Y2H Gold strain co-transformed with pGBKT7-53 and pGADT7-T was used as a positive control, and the Y2H Gold strain co-transformed with pGBKT7-lam and pGADT7-T was used as a negative control. The interaction between the tested strains was verified on SD/Leu-Trp, SD/Leu-Trp-His-Ade and SD/Leu-Trp-His-Ade containing 20 mg/mL X-α-gal agar medium. The experimental protocol used was previously described (Ma et al., 2020). This assay was performed three times.

### 2.15. Luciferase complementation assay

The *aopU* coding sequence was inserted into the pCAMBIA1300-Nluc (pNL) vector to generate pNL-AopU, and the full-length cDNA of *ClE3R* was inserted into the pCAMBIA1300-Cluc (pCL) vector to generate pCL-ClE3R (Chen et al., 2008). The constructed recombinant plasmids were transformed into *A. tumefaciens* competent cells (GV3101) respectively to obtain the corresponding *A. tumefaciens* strains. The *A. tumefaciens* strain transformed with the empty vector (pNL or pCL) was used as a control. The strains to be tested were cultured to OD_600_ = 1.0 and mixed at a 1:1 volume ratio. The mixed strains were injected into different parts of the same *N. benthamiana* leaves, and 6–9 leaves were injected each time. Samples were taken at 48 hpi, and the interaction between proteins was detected using a CCD imaging system (NightSHADE LB985; Berthold, Bad Wildbad, Germany). This assay was performed three times.

### 2.16. GST pull-down assay

The *aopU* coding sequence was inserted into the pET28a vector to generate AopU-His, and the full-length cDNA of *ClE3R* was inserted into the pGEX6P-1 vector to generate ClE3R-GST. Subsequently, AopU-His and ClE3R-GST were expressed in *E. coli* strain BL21 and purified. For GST pull-down assays, an equal amount of purified AopU-His and ClE3R-GST proteins were mixed and incubated at 4°C for 6 h. Subsequently, the mixture was loaded onto a glutathione agarose column. After washing five times, proteins were eluted with wash buffer supplemented with 15 mM reduced glutathione. Western blotting was used to analyze the eluted products as previously described (Zhang et al., 2020a). This assay was performed three times.

### 2.17. Statistical analyses

Data were analyzed by one-way analysis of variance (ANOVA) or two-way ANOVA to determine any significant differences. For RT-qPCR, data were analyzed using an independent samples t-test. *p* < 0.05 was considered statistically significant. Statistical analysis and graph generation were performed using GraphPad Prism 7.0 (GraphPad Software Inc., La Jolla, California, United States).

## 3. Results

### 3.1. Sequence analysis of the candidate T3E AopU from *Acidovorax citrulli*

**Figure 1:**
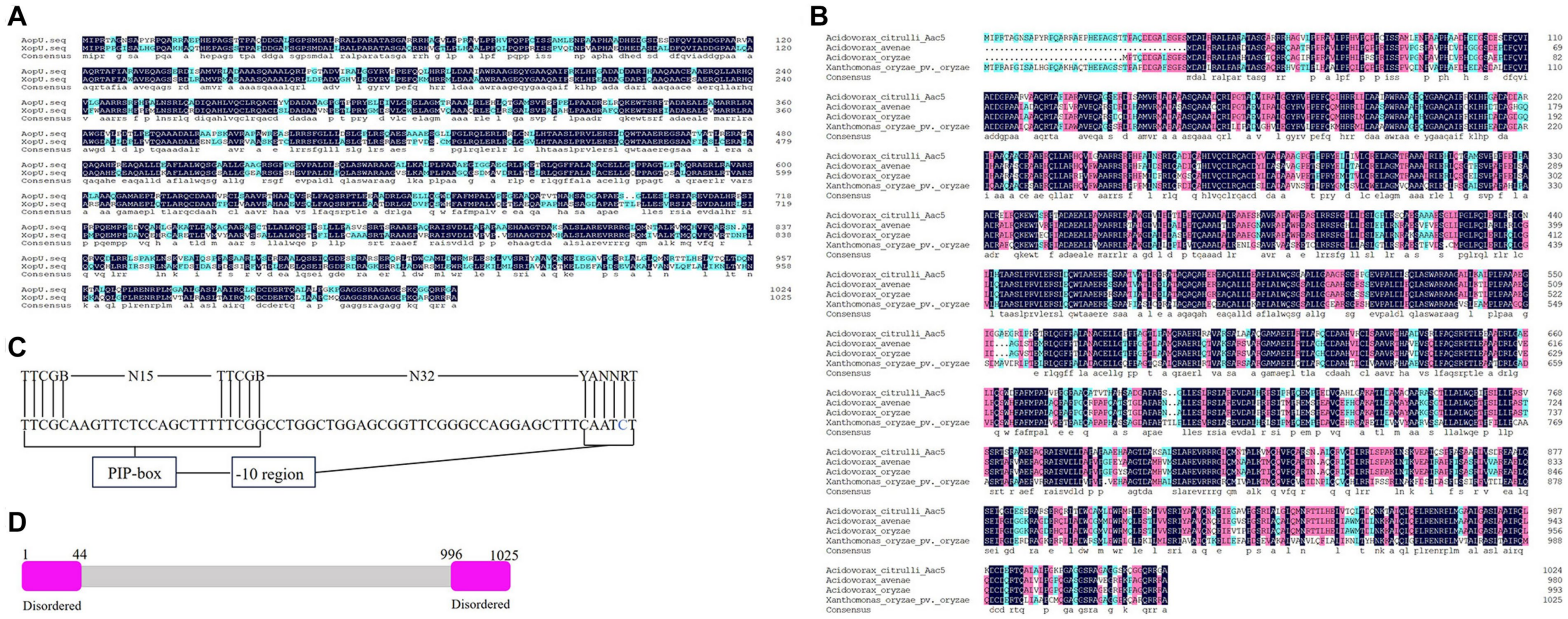
Sequence analysis of AopU in *Acidovorax citrulli*. **(A)** Amino acid sequence alignment of AopU (GenBank accession number ABM30726.1) from *A. citrulli* and XopU (GenBank accession number AEQ96632.1) from *Xanthomonas oryzae* pv. *oryzicola*. **(B)** Multiple sequence alignment of AopU and its homologous proteins from *A. avenae* (GenBank accession number WP_225975985.1), *A. oryzae* (WP_020424746.1), and *X. oryzae* pv. *oryzae* (GenBank accession number ACD58234.1). The homology level was highlighted with different colors. Dark blue represents 100% homology, pink represents homology level ≥ 75%, light blue represents homology level ≥ 50%, and the dot represents gaps. **(C)** Sequence analysis of the plant-inducible promoter (PIP-box) in the *aopU* promoter region. The first row is the consensus sequence of Gram-negative bacteria PIP-box, and the second row is the PIP-box sequence of *aopU*. The base abbreviations A, T, C, G, B, N, Y, and R were used. **(D)** The predicted structure of AopU. Both the N-terminal and C-terminal of AopU were predicted to have a disordered region.

A candidate T3E protein in *A. citrulli* group II strain Aac5 contains 1,025 amino acids, with a predicted molecular weight of 109 kDa. Its coding gene is *Aave_0114* in *A. citrulli* group II strain AAC00-1 (GenBank accession number ABM30726.1), and sequence analysis showed that the protein had homology with effector XopU (GenBank accession number AEQ96632.1) in *X. oryzae* pv. *oryzicola* (*Xoc*), so we named it AopU. The amino acid sequence alignment of AopU and XopU was shown in Figure 1A. The homologous proteins of AopU were also found in *A. avenae* (GenBank accession number WP_225975985.1), *A. oryzae* (GenBank accession number WP_020424746.1), and *X. oryzae* pv. *oryzae* (GenBank accession number ACD58234.1; Figure 1B). Notably, there is a plant inducible promoter (PIP-box, a binding site of *hrpX*) in the promoter region of the *aopU* gene (Figure 1C), and disordered regions at the N-terminal and C-terminal of AopU (Figure 1D). These are signatures of phytobacterial T3Es.

### 3.2. AopU is a T3E protein in *Acidovorax citrulli* Aac5

**Figure 2:**
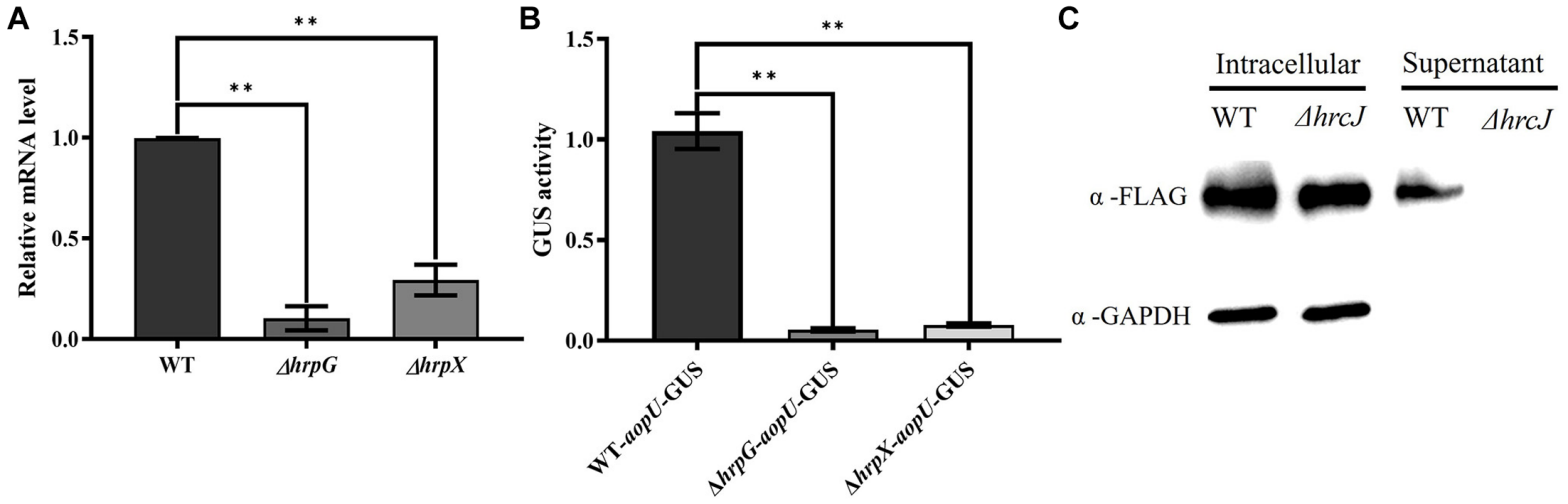
Identification of AopU as a type III effector (T3E) in *Acidovorax citrulli* Aac5. **(A)** The expression level of *aopU* in Aac5, Δ*hrpG*, and Δ*hrpX* was detected using real-time quantitative PCR. *rpoB* was used as an internal reference gene. The error bar represents the standard deviation (*n* = 3). The “**” above the bar indicates a significant difference determined by the *t*-test (*p* < 0.01). **(B)** The expression level of *aopU* in Aac5, Δ*hrpG*, and Δ*hrpX* was detected by measuring the β-Glucuronidase (GUS) activity. The promoter sequence of *aopU* fused with GUS was transferred into Aac5, Δ*hrpG*, and Δ*hrpX*, and the GUS activity in each strain was detected. The error bar represents the standard deviation (*n* = 8). The “**” above the bar indicates a significant difference determined by the *t*-test (*p* < 0.01). **(C)** The secretion of AopU depends on the type III secretion system. The intracellular and extracellular components of the tested strains WT-AopU-FLAG and *ΔhrcJ*-AopU-FLAG were extracted after being cultured in XVM2 medium, and AopU-FLAG was detected by western blot using FLAG antibody. GAPDH was used as a reference protein.

The candidate effector protein AopU from Aac5 was characterized at both the transcriptional and translation levels. Firstly, we detected the expression level of *aopU* in wild-type strain Aac5, *hrpG* mutant strain Δ*hrpG*, and *hrpX* mutant strain Δ*hrpX* using RT-qPCR. The expression of *aopU* was significantly lower in Δ*hrpG* and Δ*hrpX* compared to the wild-type (WT) strain (Figure 2A). Furthermore, we found that the GUS activity driven by the *aopU* promoter in Δ*hrpG* and Δ*hrpX* was significantly down-regulated compared to the WT (Figure 2B). These findings indicated that *aopU* is regulated by the T3SS in Aac5.

Subsequently, western blot assays were used to detect whether AopU was secreted in a T3SS-dependent manner in strain Aac5. The result showed that AopU-FLAG could be detected in the cell lysates of both the WT and Δ*hrcJ* strains, while in the supernatant, the signal was only present in WT (Figure 2C). These results provide further evidence that AopU is a T3E in *A. citrulli*.

### 3.3. *aopU* does not affect the virulence of *Acidovorax citrulli* Aac5

**Figure 3:**
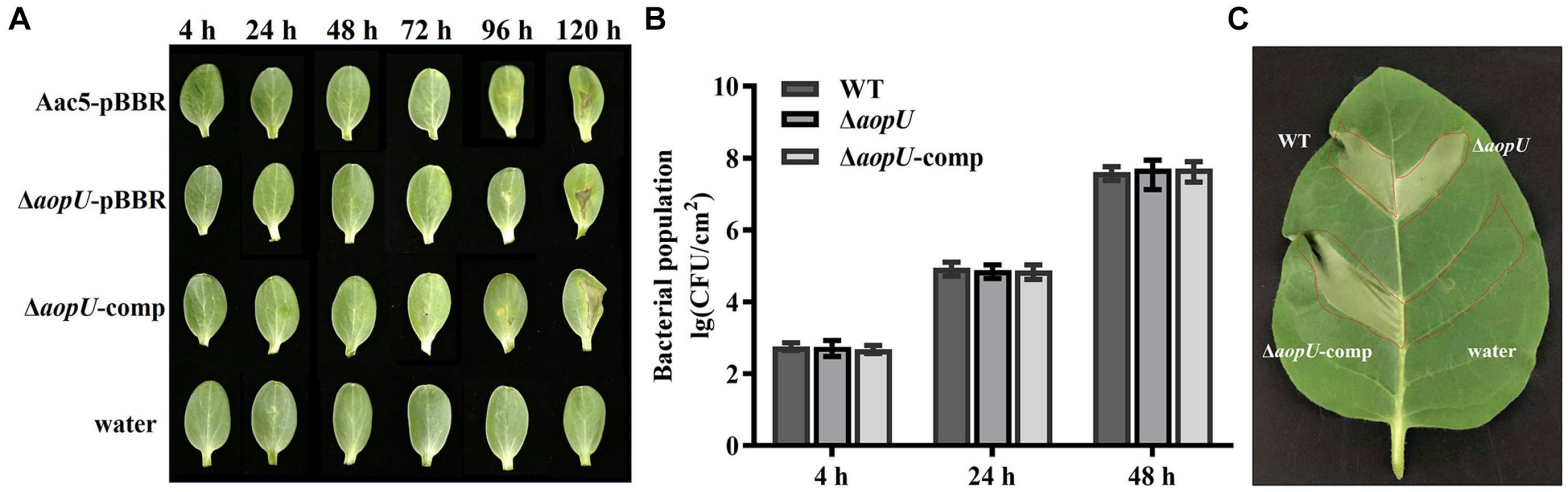
The effect of *aopU* on *Acidovorax citrulli* Aac5 virulence. **(A)** Symptoms of watermelon cotyledons after inoculation with tested strains. The bacteria growing in the logarithmic phase were centrifuged and resuspended with sterile water. Aac5-pBBR, Δ*aopU*-pBBR, and Δ*aopU*-comp were injected into watermelon at 10^4^ CFU/mL, with sterile water as a control. Symptoms were observed and pictures were taken at 4, 24, 48, 72, 96, and 120 h post inoculation (hpi). **(B)** Bacterial proliferation ability in watermelon cotyledons. Watermelon cotyledons were inoculated with Aac5-pBBR, Δ*aopU*-pBBR, and Δ*aopU*-comp at 10^4^ CFU/mL, with sterile water as a control, and sampled at 4, 24, and 48 hpi. The population levels of each strain were quantitatively analyzed. The bars represent the standard deviation of the average values of three experiments. **(C)** Hypersensitive response on non-host tobacco induced by test strains. Aac5-pBBR, Δ*aopU*-pBBR, and Δ*aopU*-comp were injected into tobacco leaves at 3 × 10^8^ CFU/mL, and the photo was taken at 48 hpi.

To investigate the role of *aopU* in Aac5 virulence, we injected strains Aac5-pBBR, Δ*aopU*-pBBR, and Δ*aopU*-comp into watermelon cotyledons. Sterile water was used as a control. Symptoms of cotyledons were observed every 24 h until 120 hpi. As shown in Figure 3A, the leaves began to turn yellow at 72 hpi, and the spots started to appear at 96 hpi. However, there was no obvious difference in symptoms among treatments of different strains. Additionally, there was no significant difference in the population levels among these strains (Figure 3B). Further, we inoculated Aac5-pBBR, Δ*aopU*-pBBR, Δ*aopU*-comp, and sterile water into the non-host tobacco to test for HR induction by these strains. The deletion of *aopU* did not affect the HR on non-host tobacco (*N. tabacum*; Figure 3C). These results implied that *aopU* does not play a noticeable role in Aac5 virulence. There may be functional redundancy among T3Es of *A. citrulli*, which may explain the lack of effect observed when *aopU* was deleted (Alfano and Collmer, 2004).

### 3.4. AopU inhibits the ROS burst in host plants

**Figure 4:**
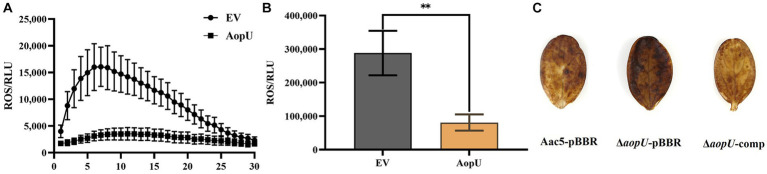
AopU suppresses the reactive oxygen species (ROS) burst in host plants. **(A)** AopU suppresses flg22-induced ROS burst in *Nicotiana benthamiana*. *Agrobacterium tumefaciens* strain GV3101 carrying pYBA1132-AopU-GFP was infiltrated into *N. benthamiana* leaves at OD_600_ = 0.5, and the GV3101 strain with the empty vector (EV) was used as the control. Samples were taken at 48 h post-inoculation (hpi) and treated with 100 nM flg22, and then the ROS amounts were detected using a multimode plate reader at 2 min intervals for 60 min. The bars represent standard errors of the means (*n* = 12). **(B)** The total amount of ROS produced in the leaves of each treatment in **(A)**. The “**” above the bar indicates a significant difference determined by the *t*-test (*p* < 0.01). **(C)** Detection of ROS accumulation using 3, 3′-diaminobenzidine (DAB) staining. Strains Aac5-pBBR, Δ*aopU*-pBBR, and Δ*aopU*-comp were injected into watermelon cotyledons at OD_600_ = 0.3. At 24 hpi, the watermelon cotyledons were sampled, stained with DAB, decolorized with 95% ethanol, and photographed.

To assess the impact of AopU on plant immunity, its effect on ROS burst in host plants was examined quantitatively and qualitatively. Our findings showed that ROS induced by flg22 was significantly increased in *N*. *benthamiana* leaves transiently expressing EV, but there was no significant change in leaves transiently expressing AopU (Figure 4A). Moreover, the total amount of ROS in the former was also significantly higher than that in the latter (Figure 4B). The qualitative test using DAB staining showed that watermelon cotyledons injected with Δ*aopU*-pBBR produced more ROS than those injected with Aac5-pBBR and Δ*aopU*-comp strains (Figure 4C). The ROS burst is generally regarded as one of the markers of PTI response (Boller and He, 2009), thus indicating that AopU can inhibit the PTI response of plants by suppressing the outbreak of ROS.

### 3.5. AopU inhibits the expression of PTI maker genes in *Nicotiana benthamiana*

**Figure 5:**
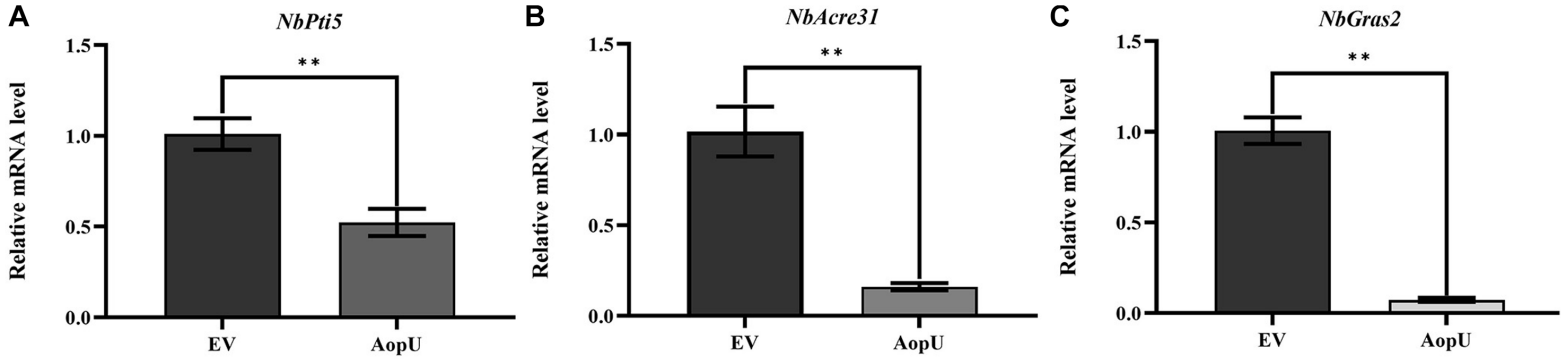
Transient expression of AopU suppresses the expression of PTI marker genes. *Agrobacterium tumefaciens* strain GV3101 carrying pYBA1132-AopU-GFP was infiltrated into *Nicotiana benthamiana* leaves at OD_600_ = 0.5, and GV3101 carrying pYBA1132-GFP was used as the negative control. Samples were taken at 48 h post-inoculation (hpi) and treated with 100 nM flg22. The total RNA of *N. benthamiana* leaves under different treatments was extracted and then the expression levels of PTI marker genes **(A)** *NbPti5*, **(B)** *NbAcre31*, and **(C)** *NbGras2* were quantitatively detected using real-time quantitative PCR. *EF1α* was used as a reference gene. The bars represent the mean with standard deviation (*n* = 3). The “**” indicates a significant difference determined by the *t*-test (*p* < 0.01).

Previous studies have shown that genes *NbPti5*, *NbAcre31*, and *NbGras2* in *N*. *benthamiana* are up-regulated when induced by PAMP, so they were used as PTI reporter genes in *N*. *benthamiana* (Nguyen et al., 2010). In this study, we examined the expression levels of these three genes in *N*. *benthamiana* leaves. These leaves were transiently expressing AopU and EV, and were treated with flg22 at 48 hpi. Compared with the control, the expression levels of PTI marker genes *NbPti5* (Figure 5A), *NbAcre31* (Figure 5B), and *NbGras2* (Figure 5C) were significantly decreased in leaf tissues transiently expressing AopU, indicating that AopU can inhibit flg22-induced PTI.

### 3.6. AopU triggers SA and JA pathways

**Figure 6:**
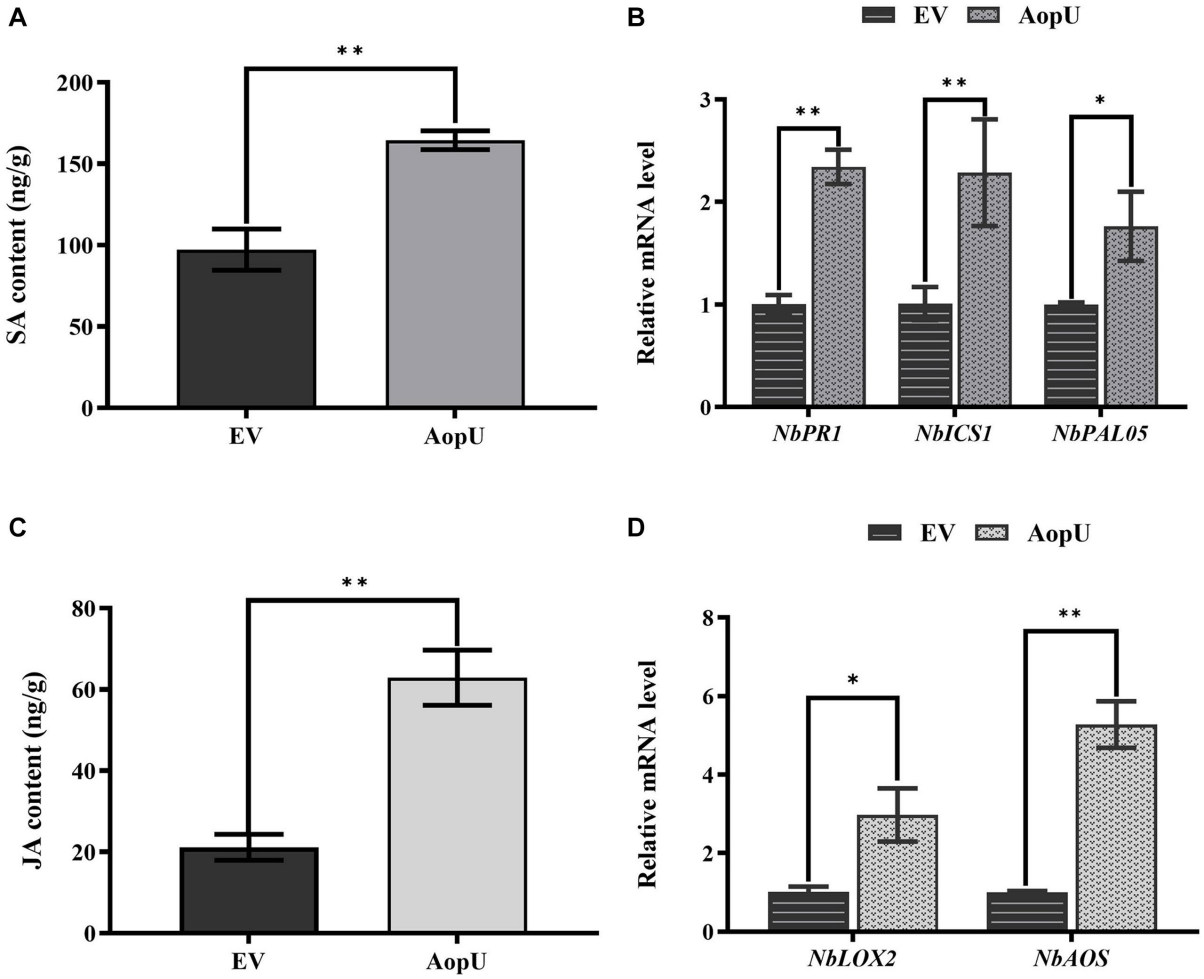
Effects of AopU on salicylic acid (SA) and jasmonic acid (JA) pathways in *Nicotiana benthamiana*. *Agrobacterium tumefaciens* strain GV3101 expressing AopU or EV was infiltrated into *N. benthamiana* leaves at OD_600_ = 0.5. Samples were collected at 48 h post-inoculation (hpi), then SA content **(A)** and JA content **(C)** in leaves were measured using high-performance liquid chromatography. The bars represent the means with standard deviation (*n* = 3). The “**” indicates a significant difference determined by the *t*-test (*p* < 0.01). RNA was extracted from leaves and reverse-transcribed into cDNA. The relative expression levels of SA marker genes **(B)** and JA marker genes **(D)** were measured using real-time quantitative PCR, with *EF1α* as the reference gene. The bars represent the means with standard deviation (*n* = 3). “*” represents *p* < 0.05, and “**” represents *p* < 0.01.

To further investigate the impact of AopU on the SA and JA signaling pathways in *N. benthamiana*, we conducted experiments and analyzed the results. The expression of AopU led to an increase in SA levels in tobacco plants (Figure 6A). Transient expression of AopU in *N. benthamiana* leaves resulted in a significant up-regulation of *NbICS1* (a key gene involved in SA synthesis), *NbPR1* (a hallmark gene of SA-mediated defense), and *NbPAL05* (a gene mediating SA synthesis; Figure 6B). SA and JA signal pathways are generally considered to be antagonistic in plant immune response (Sang et al., 2020). Surprisingly, we found that the expression of AopU also led to an increase in JA levels (Figure 6C) and upregulated expression of JA biosynthesis-related genes (*NbLOX2* and *NbAOS*) in *N. benthamiana* leaves (Figure 6D).

### 3.7. AopU localized in the cell membrane and nucleus of *Nicotiana benthamiana*

**Figure 7:**
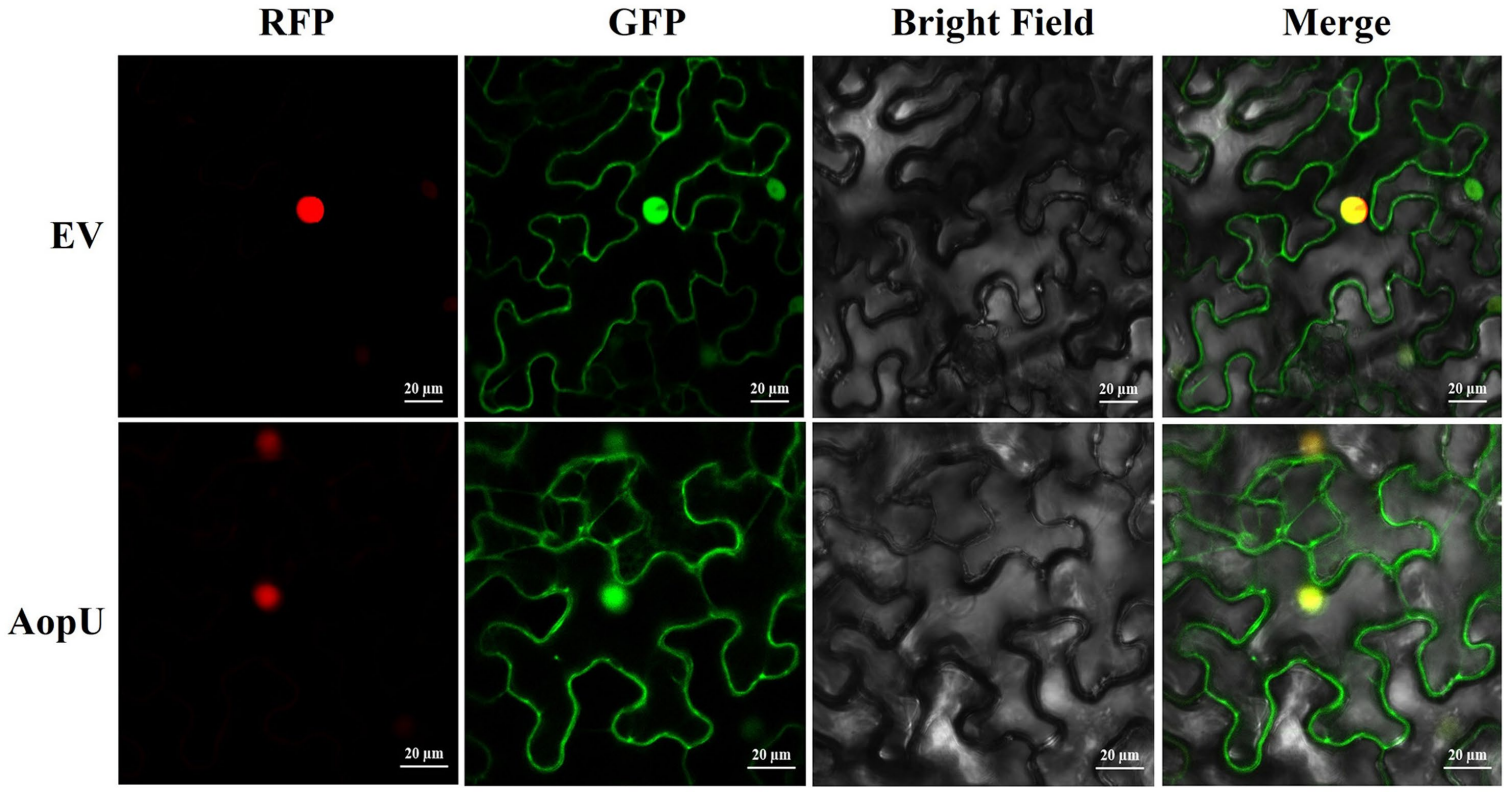
Subcellular localization of AopU. The suspensions of *Agrobacterium tumefaciens* carrying AopU-GFP and H2B-RFP were mixed in equal volumes at OD_600_ = 0.5 and then infiltrated into *Nicotiana benthamiana* leaves. The suspension of the empty vector (EV) mixed with H2B-RFP was used as a control. Samples were taken at 48 h post-inoculation (hpi) and observed using confocal microscopy at 20× magnification. “RFP” represents the red fluorescent signal emitted by the nuclear marker H2B-RFP, “GFP” represents the green fluorescent signal emitted by AopU-GFP, “Merge” represents the image of two fluorescent signals superimposed, and the yellow color indicates the overlap of green and red fluorescence. The scale bar represents 20 μm.

The localization of the effector protein in the host is closely related to its function. We transiently expressed AopU-GFP in *N. benthamiana* leaves and observed its subcellular localization using a confocal microscope. The empty vector pYBA1132-GFP was injected as a control and H2B-RFP was used as a nuclear localization marker. Fluorescent signals of AopU-GFP were observed in the cell membrane and nucleus, indicating that AopU can be expressed in both the nucleus and cytomembrane after being injected into the host (Figure 7).

### 3.8. AopU interacts with the putative E3 ubiquitin ligase ClE3R in watermelon

**Figure 8:**
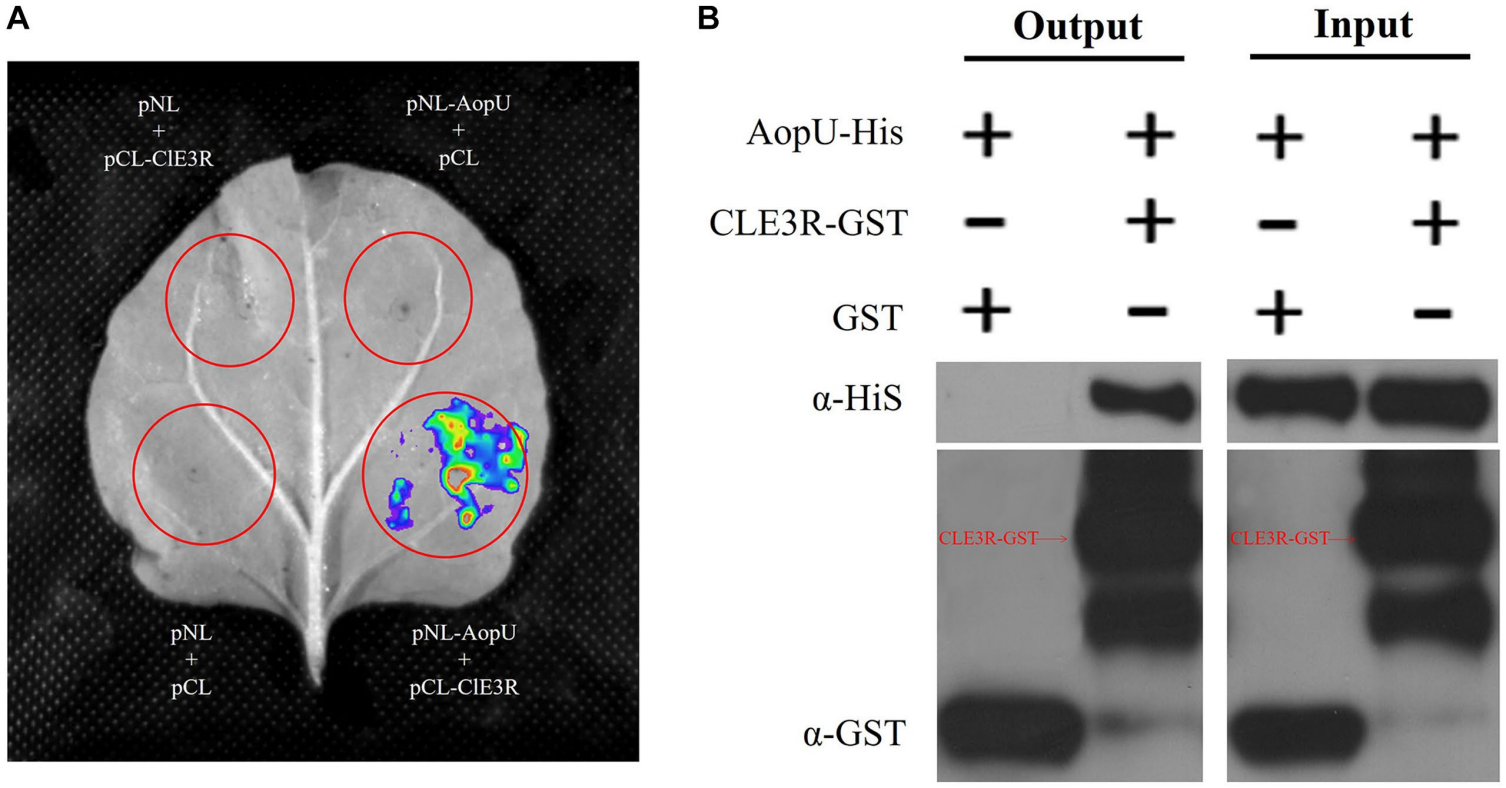
Identification of the interaction between AopU and ClE3R. **(A)** Luciferase complementation assay (LCA) to verify the interaction. *Agrobacterium tumefaciens* strains carrying pNL-AopU + pCL-ClE3R, pNL-AopU + pCL, pNL + pCL-ClE3R, and pNL + pCL were injected into different positions of the same *Nicotiana benthamiana* leaves. Leaves were analyzed using a CCD imaging system at 48 hpi. Fluorescence signals could be captured at the site injected with pNL-AopU + pCL-ClE3R, but not at control sites. **(B)** Glutathione S-Transferase (GST) pull-down assay to verify the interaction. The purified AopU-His protein was incubated with ClE3R-GST and GST purified protein, respectively, and precipitated using glutathione agarose. Immunoblotting of input proteins and pull-down proteins was performed using anti-His and anti-GST antibodies.

To better understand the mechanism of AopU interfering with the host immune response, we conducted a screening process to identify its interaction proteins in watermelon. By using AopU as bait, we successfully screened and identified a target protein predicted to be E3 ubiquitin ligase MIEL1 from the watermelon cDNA library using the yeast two-hybrid assay (Supplementary Figure S1). This protein is encoded by *Cla014698* in watermelon (97103) genome and contains a RING finger domain, named ClE3R (Supplementary Figure S2). The interaction between AopU and ClE3R was further verified using a luciferase complementation assay. Two days after co-expression of pNL-AopU and pCL-ClE3R in *N. benthamiana*, the fluorescent signal was observed at the injection site using the CCD imaging system, but not in the control treatments pNL-AopU + pCL, pNL + pCL-ClE3R, and pNL + pCL (Figure 8A). Additionally, the GST-pull-down assay was also employed to verify the interaction. The purified AopU-His protein was incubated with glutathione agarose columns with purified ClE3R-GST and GST proteins respectively, and the eluted products were detected by western blot. The results demonstrated that AopU could precipitate with ClE3R-GST, but not with GST (Figure 8B). These results suggested that AopU and ClE3R can interact directly both *in vivo* and *in vitro*.

## 4. Discussion

T3Es are key factors in the pathogenesis of *A. citrulli* (Jiménez-Guerrero et al., 2020). However, due to the challenges in genetically manipulating watermelon, studies on the mechanism of action of *A. citrulli* T3Es have fallen behind those of *Xanthomonas* and *Pseudomonas*. Nonetheless, recent advancements in the identification system for *A. citrulli* effectors and the use of *Nicotiana* species as alternative hosts have greatly facilitated the characterization of *A. citrulli* T3Es (Traore et al., 2019; Zhang et al., 2020b). In this study, we found that AopU from *A. citrulli* group II strain Aac5 was homologous to the effector XopU from *Xoc*. Sequence analysis showed that the homologous proteins of AopU in *Xanthomonas* are only found in *Xoo* and *Xoc*, which is consistent with a previous report stating that XopU is unique to *X. oryzae* (Song and Yang, 2010). This suggests that the protein may only function in specific species, possibly due to bacterial evolution. Previous studies have shown that in *A. citrulli*, *hrpG* activates the expression of *hrpX*, which in turn regulates the expression of certain T3E genes by binding to the PIP box (Zhang et al., 2018). In the promoter region of AopU, a perfect PIP-box is located 89 bp upstream of the initiation codon ATG. Further experiments have confirmed that AopU is indeed a protein regulated by *hrpG* and *hrpX*, and its secretion depends on the T3SS. Therefore, we have successfully identified a novel effector protein in Aac5.

Elucidating the functions of individual T3Es is an important initial step in understanding their respective contributions to virulence (Deslandes and Rivas, 2012). We investigated the function of AopU and showed that it could inhibit the PTI response in *N. benthamiana* by suppressing the ROS burst and the expression of certain PTI marker genes. This finding is consistent with the previously identified functions of other effectors (AopN, AopP, and AopV) from *A. citrulli*. Notably, AopU was able to activate both SA and JA signal pathways, as evidenced by increased levels of SA or JA and the upregulation of SA or JA marker genes in *N*. *benthamiana* leaves transiently expressing AopU.

It is well-established that SA and JA play crucial roles as phytohormones in plant defense. SA primarily mediates plant resistance against biotrophic and hemibiotrophic pathogens, whereas JA primarily mediates resistance against necrotrophic pathogens (Robert-Seilaniantz et al., 2007). Some plant pathogenic bacteria have evolved effectors to manipulate these defense mechanisms. Typically, SA and JA pathways are antagonistic, and certain phytopathogenic bacteria such as *P. syringae* and *Xanthomonas* spp. have evolved effectors to manipulate the resistance regulated by these phytohormones (Mudgett, 2005; Kazan and Lyons, 2014). For example, XopD in *X. campestris* was shown to inhibit SA signaling during bacterial infection of tomato (Kim et al., 2008), while HopZ1 in *P. syringae* indirectly inhibits SA accumulation by activating JA signaling (Jiang et al., 2013). However, very few T3Es activate both SA and JA signaling pathways simultaneously. Interestingly, the relationship between SA and JA is not always antagonistic. There is evidence of a synergistic interaction between SA and JA during ETI, with the endogenous JA level increasing along with SA accumulation (Liu et al., 2016). Therefore, we hypothesize that AopU may have triggered ETI. However, different plant species may have varying requirements for these two pathways in their immune responses (Sang et al., 2020), and further research is needed to understand the specific mechanism by which AopU regulates SA and JA.

Regarding the role of AopU in virulence, deleting *aopU* does not affect *A. citrulli* virulence in watermelon, similar to the case of XopU (Song and Yang, 2010). Furthermore, a previous study found that deleting *aopN* also did not affect *A. citrulli* virulence in watermelon. This suggests that *A. citrulli* may employ other functional redundant effectors during the infection of watermelon (Zhang et al., 2020b). In fact, effectors usually make an additive contribution to bacterial virulence in host plants. Therefore, the deletion of a single T3E gene may not have any noticeable effects on susceptible plants (Traore et al., 2019). Moreover, certain effectors can induce lesions in the host without affecting bacterial growth (Alfano and Collmer, 2004). In addition, the same effector protein may exhibit varying degrees of virulence in different strains or on different hosts, further complicating the characterization of the T3E function (Zhang et al., 2020b).

The ubiquitin proteasome system (UPS) is a mechanism that regulates various cellular processes in plants and plays an important role in plant immunity (Vierstra, 2009). The ubiquitination process involves a cascade reaction of the E1 ubiquitin-activating enzyme, E2 ubiquitin-conjugating enzyme, and E3 ubiquitin ligase (Vierstra, 2003). Among them, E3 ubiquitin ligase specifically recognizes target proteins. E3 ubiquitin ligases are categorized into four classes based on their structural domains: homologous to E6-associated protein C-terminus (HECT), U-box, really interesting new gene (RING), and cullin-RING ligases (CRLs; Vierstra, 2009). Numerous studies have shown the involvement of E3 ubiquitin ligases in regulating plant defense response. For instance, the U-box E3 ubiquitin ligases AtPUB22, AtPUB23, and AtPUB24 negatively regulate PTI in *A. thaliana* (Trujillo et al., 2008). In addition, the U-box protein OsSPL11 in rice regulates cell death and plant defense (Li et al., 2012).

E3 ubiquitin ligases are also important targets for T3Es. The effector AvrPiz-t of *M. oryzae* inhibits PTI in rice by targeting the RING E3 ubiquitin ligase APIP6 (Park et al., 2012). Similarly, XopP_Xoo_ directly interacts with the U-box structural domain of the rice E3 ubiquitin ligase OsPUB44 and inhibits its ligase activity (Ishikawa et al., 2014). In this study, we screened target proteins of AopU in watermelon and identified an interaction between CLE3R and AopU. Amino acid sequence analysis revealed high homology of CLE3R to the RING E3 ubiquitin ligase MIEL1. Previous studies have reported that MIEL1 can negatively regulate the JA pathway (An et al., 2021). Furthermore, MIEL1 mediates the degradation of the transcription factor MYB30, which leads to a weakened plant defense response in *Arabidopsis* (Marino et al., 2013). Based on these findings, we speculate that AopU may manipulate the host’s immune responses by targeting E3 ubiquitin ligase, thereby modulating the PTI, SA, and JA signaling pathways.

Currently, the functional description of *X. oryzae* XopU is limited to the inhibition of XopQ-XopX-induced immune response. In this study, we investigated the function of its homologous protein AopU in *A. citrulli* group II strain Aac5. We also discovered the interaction between AopU and the putative E3 ubiquitin ligase CLE3R in the host watermelon. However, further experiments are required to clarify the response of CLE3R to the immune response of watermelon and its involvement in the SA and JA pathways. In addition, we are also interested in exploring the potential interaction between AopU and MIEL1 in *N*. *benthamiana*. Currently, we have observed the localization of AopU in the cell membrane and nucleus in *N*. *benthamiana*, but its functional relevance remains unknown. Nonetheless, this study provides a new avenue for uncovering the mechanism of interactions between *A. citrulli* T3Es and their host plants.

## Data availability statement

The datasets presented in this study can be found in online repositories. The names of the repository/repositories and accession number(s) can be found in the article/Supplementary material.

## Author contributions

LY: Conceptualization, Data curation, Formal analysis, Methodology, Resources, Validation, Visualization, Writing – original draft, Writing – review & editing. MZ: Formal analysis, Writing – review & editing. XZ: Conceptualization, Methodology, Writing – review & editing. JJ: Validation, Writing – review & editing. NF: Validation, Writing – review & editing. WJ: Validation, Writing – review & editing. YY: Formal analysis, Writing – review & editing. WG: Formal analysis, Writing – review & editing. YY: Supervision, Writing – review & editing, Formal analysis. TZ: Conceptualization, Funding acquisition, Supervision, Writing – review & editing.

## Footnotes

^1^ https://www.ncbi.nlm.nih.gov/

^2^ https://www.uniprot.org/
