# Supplementary figures and images for: Acidovorax citrulli type III effector AopU interferes with plant immune responses and interacts with a watermelon E3 ubiquitin ligase
Source: Front Microbiol. 2023 Oct 9;14:1275032. doi: 10.3389/fmicb.2023.1275032 (PMC10590900; doi:10.3389/fmicb.2023.1275032)

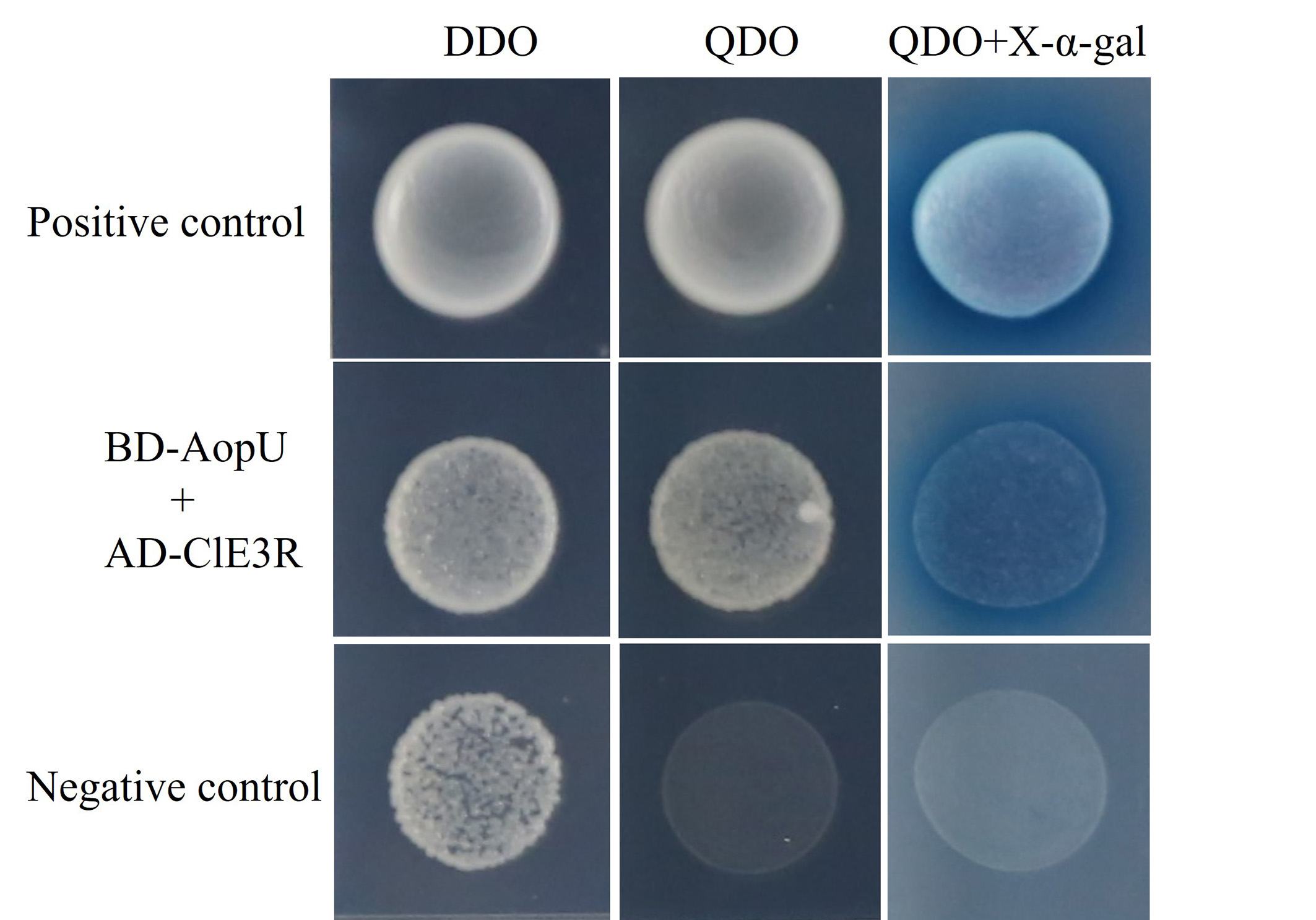

Supplement: Supplementary file 1 [file Image_1.JPEG]

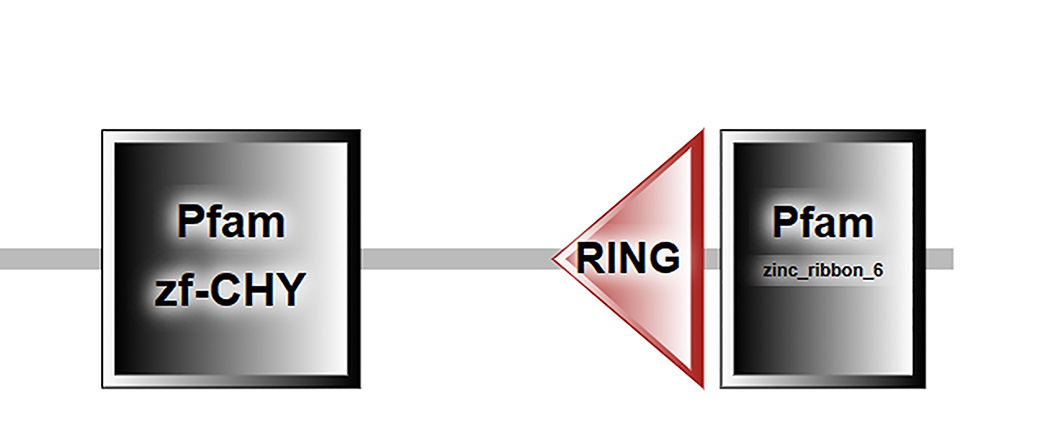

Supplement: Supplementary file 2 [file Image_2.JPEG]
