# Supplementary material for: Acidovorax citrulli type III effector AopU interferes with plant immune responses and interacts with a watermelon E3 ubiquitin ligase
Source: Front Microbiol. 2023 Oct 9;14:1275032. doi: 10.3389/fmicb.2023.1275032 (PMC10590900; doi:10.3389/fmicb.2023.1275032)
Supplement: Supplementary file 3 [file Table_1.docx]

| **Table S1 Bacterial strains and plasmids used in this study** | | |
| --- | --- | --- |
| **Strains and plasmids** | **Description** | **Reference or source** |
| Strains |  |  |
| *Acidovorax citrulli* |  |  |
| Aac5 | Wild-type group II strain, Amp^r^ | Lab collection |
| Aac5-pBBR | Aac5 containing pBBRNolac-4FLAG, Amp^r^, Kan^r^ | This study |
| Δ*hrpG* | *hrpG* markerless mutation of Aac5, Amp^r^ | This study |
| Δ*hrpX* | *hrpX* markerless mutation of Aac5, Amp^r^ | This study |
| *ΔhrcJ* | *hrcJ* markerless mutation of Aac5, Amp^r^ | This study |
| Δ*aopU* | *aopU* markerless mutation of Aac5, Amp^r^ | This study |
| Δ*aopU*-pBBR | Δ*aopU* containing pBBRNolac-4FLAG, Amp^r^, Kan^r^ | This study |
| Δ*aopU*-comp | Δ*aopU* containing pBBRNolac-*aopU*-FLAG | This study |
| WT-*aopU*-GUS | Aac5 containing pBBR-*aopU*-GUS, Amp^r^, Km^r^ | This study |
| Δ*hrpG*-*aopU*-GUS | Δ*hrpG* containing pBBR-*aopU*-GUS, Amp^r^, Km^r^ | This study |
| Δ*hrpX*-*aopU*-GUS | Δ*hrpX* containing pBBR-*aopU*-GUS, Amp^r^, Km^r^ | This study |
| WT-*aopU*-FLAG | Aac5 containing pBBRNolac-*aopU*-FLAG, Amp^r^, Km^r^ | This study |
| Δ*hrcJ*-*aopU*-FLAG | Δ*hrcJ* containing pBBRNolac-*aopU*-FLAG, Amp^r^, Km^r^ | This study |
| *Escherichia coli* |  |  |
| DH5α | *supE44 ΔlacU169(Φ80lacZ ΔM15) hsdR17 recA1 endA1 gyrA96 thi-1 relA1* | TIANGEN, Beijing, China |
| *Agrobacterium tumefaciens* |  |  |
| GV3101 | *C58 (rif^r^) Ti pMP90 (pTiC58DT-DNA) (gentr/strepr) Nopaline* | BIOMED, Beijing, China |
| Plasmids |  |  |
| pK18mobsacB | Suicide vector with *sacB* gene, Km^r^ | Lab collection |
| pK18-*aopU* | Suicide vector containing upstream and downstream fragments of *aopU* gene on pK18mobsacB, Km^r^ | This study |
| pBBR-*aopU* | pBBRNolac-4FLAG carrying *aopU* and its native promoter, Km^r^ | This study |
| pBBRNolac-GUS | *lac* promoter was deleted from pBBR1MCS-2 and GUS reporter gene was inserted, Km^r^ | Zhang et al., 2018 |
| pBBR-*aopU*-GUS | pBBRNolac-GUS carrying the native promoter of *aopU*, Km^r^ | This study |
| pBBRNolac-4FLAG | *lac* promoter was deleted from pBBR1MCS-2 and C-terminal 4×FLAG tag was inserted, Km^r^ | Zhang et al., 2018 |
| pBBRNolac-*aopU*-FLAG | pBBRNolac-4FLAG carrying *aopU* with its native promoter, Km^r^ | This study |
| pRK600 | Helper strain in tri-parental mating, Cm^r^ | Lab collection |
| pYBA1132 | Plant expression vector containing a 35S promoter and eGFP tag, Kan^r^ | Lab collection |
| pYBA1132-*aopU* | pYBA1132 carrying *aopU* with no termination codon, Kan^r^ | This study |
| pGBKT7-AopU | pGBKT7 containing AopU for yeast two hybrid assay, Km^r^ | This study |
| pGADT7-ClE3R | pGADT7 containing ClE3R for yeast two hybrid assay, Amp^r^ | This study |
| pCAMBIA1300-nLUC | Plant expression vector containing 35S promoter, carrying nLUC tag, Km^r^ | Chen et al., 2008 |
| pCAMBIA1300-nLUC-AopU | pCAMBIA1300-nLUC fused with AopU, Km^r^ | This study |
| pCAMBIA1300-cLUC | Plant expression vector containing 35S promoter, carrying cLUC tag, Km^r^ | Chen et al., 2008 |
| pCAMBIA1300-cLUC-ClE3R | pCAMBIA1300-cLUC fused with ClE3R, Km^r^ | This study |
| pSPYNE®173 | Plant expression vector containing 35S promoter, carrrying nYFP tag, Km^r^ | Lab collection |
| pSPYNE®173-AopU | pSPYNE®173 fused with AopU, Km^r^ | This study |
| pSPYCE(M) | Plant expression vector containing 35S promoter, carrrying cYFP tag, Km^r^ | Lab collection |
| pSPYCE(M)-ClE3R | pSPYCE(M) fused with ClE3R, Km^r^ | This study |
| pET28a | Prokaryotic expression vector, carrying His_6_ tag, Km^r^ | Lab collection |
| pGEX6P-1 | Prokaryotic expression vector, carrying GST tag, Amp^r^ | Lab collection |
| pET28a-AopU | pET28a containing AopU, Km^r^ | This study |
| pGEX6P-ClE3R | pGEX6P containing ClE3R, Amp^r^ | This study |
| Amp^r^, Km^r^, and Rif^r^, and Cm^r^ indicate resistance to ampicillin, kanamycin, rifampicin, and chloramphenicol, respectively | | |
