# Supplementary material for: Acidovorax citrulli type III effector AopU interferes with plant immune responses and interacts with a watermelon E3 ubiquitin ligase
Source: Front Microbiol. 2023 Oct 9;14:1275032. doi: 10.3389/fmicb.2023.1275032 (PMC10590900; doi:10.3389/fmicb.2023.1275032)
Supplement: Supplementary file 4 [file Table_2.docx]

**Table S2 Primers used in this study.**

| **Primer name** | **Primer sequence (5’-3’)** | **Product Length** |
| --- | --- | --- |
| RT-rpoB-F | GCGACAGCGTGCTCAAAGTG | 104 bp |
| RT-rpoB-R | GCCTTCGTTGGTGCGTTTCT |  |
| RT-0114F | CTCGTTGTCAGCCGCATCTA | 146 bp |
| RT-0114R | GCCGTCTTGTTCTGGTCTGT |  |
| 0114-GusF | CGCGGTGGCGGCCGCTCTAGAGCAGGGCGTGGTTGGGAA | 551 bp |
| 0114-GusR | CATAAGCTTGATATCGAATTCGGAATGTCATTTTTTGCATGGA |  |
| 0114-FlagF | CGCTCTAGAACTAGTGGATCC GGCTGCCCGCCACCCG | 3561 bp |
| 0114-FlagR | GGTAAGCTTGATATCGAATTCTGGTGCGCCCCGGCG |  |
| Comp-0114F | ACCGGGCCCCCCCTCGAGCGCAGGGCGTGGTTGG | 3627 bp |
| Comp-0114R | GGCCGCTCTAGATGGTGCGCCCCGGC |  |
| 1132-0114F | CGCTCTAGAACTAGTGGATCCATGATTCCGCGTACCGCA | 3075 bp |
| 1132-0114R | GGGCCCCCCCTCGAGGTCGACTGGTGCGCCCCGGCG |  |
| WFB1 | GACCAGCCCACACTGGGAC | 360 bp |
| WFB2 | CTGCCGCACTCCAGCGA |  |
| Km-F | ATGATTGAACAAGATGGATTGCAC | 795 bp |
| Km-R | TCAGAAGAACTCGTCAAGAAGGC |  |
| NbPti5-F | CCTCCAAGTTTGAGCTCGGATAGT |  |
| NbPti5-R | CCAAGAAATTCTCCATGCACTCTGTC |  |
| NbAcre31-F | AATTCGGCCATCGTGATCTTGGTC |  |
| NbAcre31-R | GAGAAACTGGGATTGCCTGAAGGA |  |
| NbGras2-F | TACCTAGCACCAAGCAGATGCAGA |  |
| NbGras2-R | TCATGAGGCGTTACTCGGAGCATT |  |
| EF1α-F | AAGGTCCAGTATGCCTGGGTGCTTGAC |  |
| EF1α-R | AAGAATTCACAGGGAC AGTTCCAATACCA |  |
| NbPR1-F | GGTCAACACGGCGAAAACC |  |
| NbPR1-R | GCCTTAGCAGCCGTCATGA |  |
| NbICS1-F | GTGTCGGCTCTGCTGTCTTCT |  |
| NbICS1-R | CTGCGTATAGCACGCCAATC |  |
| NbPAL05-F | AAGGGAGCTGAAATCGCCAT |  |
| NbPAL05-R | TCCGCACTTTGGACATGGTT |  |
| NbLOX2-F | TCTTGGGTGGCTCCTCTGACT |  |
| NbLOX2-R | TGTTGGAGGTCTGCCTGTTCT |  |
| NbAOS-F | CTGGGGTCAAACTCCACACT |  |
| NbAOS-R | TTGTGATGCAACTGGTGGTT |  |
| Nluc-0114F | ACGGGGGACGAGCTCGGTACCATGATTCCGCGTACCGCA | 3078 bp |
| Nluc-0114R | CGCGTACGAGATCTGGTCGACTCATGGTGCGCCCCG |  |
| E3RCluc-F | TACGCGTCCCGGGGCGGTACCATGCGAGCATTACAGGAGGC | 804bp |
| E3RCluc-R | ACGAAAGCTCTGCAGGTCGACTCATTGACGTCCTCCAGAAGAA |  |
| PET-0114F | AGCTTGTCGACGGAGCTCGAAATGATTCCGCGTACCGCA | 3078 bp |
| PET-0114R | GTGGTGGTGGTGGTGCTCGAGTCATGGTGCGCCCCG |  |
| 6PE3RF | TTCCAGGGGCCCCTGGGATCCATGCGAGCATTACAGGAGGC | 804 bp |
| 6PE3RR | GATGCGGCCGCTCGAGTCGACTCATTGACGTCCTCCAGAAGAA |  |
